# The theme of death in texts written by patients with endogenous mental disorders

**DOI:** 10.1192/j.eurpsy.2025.2206

**Published:** 2025-08-26

**Authors:** T. I. Medvedeva, S. N. Enikolopov, O. Y. Vorontsova, O. M. Boyko

**Affiliations:** 1 Clinical psychology, Mental Health Research Center, Moscow, Russian Federation

## Abstract

**Introduction:**

A specific attitude towards death is an important marker of suicidal risk. A freely written text can be considered as a variant of a projective test and can assess implicit attitudes toward death and suicide.

**Objectives:**

The **objective** was to identify the psychological characteristics of the subjects using the theme of death in the texts.

**Methods:**

91 patients with schizophrenic and affective spectrum disorders (43 men and 48 women). The control N=98 (40 men and 58 women). All the subjects wrote a short essay “Me, others, the world”, the presence of words from the thematic group “death” was assessed, Big Five Inventory (BFI) and SCL-90R were used. The question about the presence of suicidal thoughts was measured on a Likert scale.

**Results:**

Control and clinical groups did not differ in the frequency of the topic of death (13.3% of the control and 16.5% in clinical group). However, patients wrote more often about themselves, while in control «death» was more often encountered when discussing problems of humanity, ecology, loneliness. The subjects from the clinical group showed low: “Extraversion” (48,86±10,07 and 44,72±10,44 for the control and clinical groups) (due to low “Activity”, “Excitement seeking” and “Gregariousness”), “Conscientiousness” (“Neat” and “Decisiveness”), “Emotional Stability” (50,58±12,21 and 54,88±11,42) (due to “Tension”, “Depression” and “Self-punishing”). Сcomparison of the subgroups that mentioned «death» (control and clinical subgroups) revealed no statistical differences in BFI (clinical subgroup demonstrated a more pronounced “insensitivity”). In general, all subjects (both healthy and clinic patients) with the topic of death differed from the group of subjects who did not touch death vocabulary: “Introversion” (47.66±9.77 and 40.58±13.45 for the subgroup without «death» and with the topic of death), “Attachment” (53,25 ±10,06 and 60,25± 7,03), high “Emotionality” (48,85±11,87 and 56,91± 10,88). A similar pattern is observed when comparing the subgroups “control without the theme of death” and “control with the theme of death”. In the clinical group, it was shown that the topic of death is associated with a higher suicidal risk (the question about the severity of the intention to commit suicide, the average values are 0.30±0.57 and 1.16±1.60 for the “clinical subgroup without the topic of death” and “clinical subgroup with the topic of death”, respectively).

**Conclusions:**

The topic of death in control group indicates a conflict between introversion and social orientation, as well as an inability to control their emotions and impulsive drives and low self-esteem. In the clinical group, almost all of whose subjects differ from the healthy group by increased introversion and emotionality, mentioning the topic of death may be a marker of increased suicidal risk.

**Disclosure of Interest:**

None Declared

